# Early Drug Discovery Prediction of Proarrhythmia Potential and Its Covariates

**DOI:** 10.1208/s12248-015-9773-1

**Published:** 2015-05-05

**Authors:** Sebastian Polak, Michael K. Pugsley, Norman Stockbridge, Christine Garnett, Barbara Wiśniowska

**Affiliations:** Certara, St. Louis, Missouri USA; Simcyp (part of Certara), Sheffield, UK; Jagiellonian University Medical College, Cracow, Poland; Department of Global Safety Pharmacology & Toxicology/Pathology, Janssen Pharmaceuticals LLC, Raritan, New Jersey 08869 USA; Center for Drug Evaluation and Research, Food and Drug Administration, Silver Spring, Maryland USA

## INTRODUCTION

Cardiotoxicity remains a major concern during drug development, with increased proarrhythmic potential being the main culprit. The pharmaceutical industry is challenged by the growing cost of research and development and cannot afford drug attrition in late phases of development or withdrawals of approved drugs. The ICH S7B non-clinical and E14-based clinical methodologies have been successful in their intent to reduce and eliminate drug-mediated torsades de pointes (TdP) arrhythmias as there have not been any withdrawals of marketed drugs for torsadogenic reasons since these guidelines were adopted (1). However, the current approach is conservative and can result in false positives. Thus, while effective, the current paradigm may be inappropriately assigning TdP liability to some drugs, especially in the discovery realm, so work is ongoing to shift from one that strongly relies on QT interval prolongation (or is “hERG-centric”) to one where proarrhythmic risk would be primarily assessed using non-clinical *in vitro* human models based on solid mechanistic considerations of TdP proarrhythmia (2) in conjunction with the current *in vivo* QT conscious canine and other non-rodent models.

Problems related to this drug-induced cardiotoxicity were discussed at a symposium entitled “*A Gentle Touch on the Beating Heart*: *Early Discovery Prediction of Cardiotoxicity and Its Covariates*” and conducted during the AAPS 2014 Annual Meeting. The aim of this mini-symposium was to present methods of predicting cardiotoxicity (which will be defined throughout this entire manuscript as a synonym of a proarrhythmic effect) from early discovery data. Three speakers from different backgrounds introduced the audience to the current safety testing paradigm, the latest achievements within drug safety and remaining hurdles regarding the integration of multiple sources of data acquired both at the preclinical and clinical level, and their translation to the human *in vivo* situation. Despite a reasonable knowledge of the mechanisms related to proarrhythmic cardiotoxicity, there are still questions whether the new paradigm of testing will be able to provide all stakeholders with a sufficient level of confidence with the application of these novel concepts and models to drug safety.

This article gives a short overview of the discussed problems, summarizes the discussion, and gives a flavor of a new cardiac safety testing pathway likely to be introduced in the near future. Some of the statements in the text, like the feasibility of the individualized risk assessment, can be contradictory what was left intentionally and shows the line of discussion between speakers. The three leading topics include a description of the molecular and clinical background of drug-induced proarrhythmic effects, a brief presentation of current methods used in the early prediction of such effects together with anticipated changes in the testing paradigm and a discussion of the *in vitro*–*in vivo* extrapolation approach, all of which promise the possibility of risk assessment at the individual patient level.

### Molecular and Clinical Background of the Drug-Triggered Cardiac Arrhythmia

The development of TdP is a potentially life-threatening ventricular arrhythmia that can occur as an unintended result of drug therapy of various, in many cases relatively benign, conditions. It’s rare occurrence hinders TdP observation considerably during the conduct of clinical trials, thus necessitating the use of surrogate markers. For most medications known to cause torsades, TdP is a result of repolarization delay or alterations clinically reflected by QT interval prolongation on the surface electrocardiogram (ECG). Despite being considered an imperfect biomarker with compromised sensitivity and specificity and lack of a straightforward correlation with TdP occurrence, QT interval prolongation remains the most globally used biomarker to assess the proarrhythmic propensity of a drug (3,4). QT interval prolongation is a mechanism-based effect resulting primarily from the inhibition of rapid delayed rectifier potassium current (*I*_Kr_) mediated by ion channels encoded by the human ether-a-go-go-related gene (hERG or Kv11.1). It has been shown that *in vitro* measured *I*_Kr_ inhibition potency expressed as an IC_50_ value correlates with the clinically observed arrhythmia occurrence (5,6). This fact is reflected in the regulatory requirements, which require such *in vitro* study to be conducted before a first-in-human trial (7). Despite the importance of the hERG channel in safety pharmacology, studies investigating hERG channel exclusively might not fully reflect the drug potential in modifying cardiac ionic currents. Since the action potential of ventricular cardiomyocytes is a result of complex interplay between inward and outward ionic currents, drug interaction with an inward current can alter the effect of hERG inhibition of outward current and hence may limit effects on the QT interval and subsequent assessed proarrhythmic potential. Indeed, there are potent hERG inhibitors devoid of TdP risk (e.g., verapamil, propafenone, amiodarone, ranolazine) because of either a counteraction to potassium current inhibition by either sodium or calcium channel blockade or other potential antiarrhythmic mechanisms (8,9). On the other hand, alfuzosin is an example of a drug with negligible hERG liability although it causes QTc interval prolongation by increasing sodium current (8–11). Therefore, one of the proposed changes to be implemented into cardiac safety testing is an evaluation of the drug effects on multiple cardiac ionic currents. Recently, Kramer *et al*. (12) assessed whether determining concomitant block of multiple ion channels could more accurately predict the torsadogenic potential of a compound rather than just examining the effects on hERG alone. The application of logistic regression models using data derived from high-throughput screening multiple ion channel electrophysiology (MICE) methods showed that for the known 23 non-torsadogenic and 32 torsadogenic drugs from multiple classes tested that there was a significant reduction in false positives (i.e., type 1 errors) and false negatives (i.e., type 2 errors) when compared to assessment using the hERG assay alone.

Currently, there are efforts to develop a Comprehensive *In vitro* Proarrhythmia Assay (CIPA) that aims to modify and modernize current non-clinical, “hERG-centric” cardiac safety screening efforts (2,13,14). Current alternative screening models and methods under consideration for the CIPA initiative include stem cells in which hERG (Kv11.1; *I*_Kr_ current) as well as other cardiac ion channels such as the fast sodium (Nav1.5; *I*_Na_ current) channel, persistent sodium channel (*I*_Nasus_), calcium (Cav1.2; *I*_Ca_ current) channel as well as potassium channels such as the inward rectifier (Kir2.1-2.4; *I*_K1_ current), and slow delayed rectifying (Kv7.1; *I*_Ks_ current) channel can be assessed in totality (15). Rather than examining human ion channel isoforms heterogeneously expressed in cell lines (such as CHO or HEK) as is current practice in drug safety, there is ongoing investigation of the applicability of human-induced pluripotent stem cells (16). The undifferentiated human stem cell of embryonic origin (hESC) and induced pluripotent stem cell (iPSCs) of somatic origin continue to be evaluated for their cardiac electrophysiological potential for use as a drug screening assay (16–19). Intracellular recordings from individual cells and multi-electrode arrays (MEAs) enable measurement of sodium, calcium, and potassium current from stem cells (16). Note, however, that a limitation to the use of stem cells that are currently being evaluated is that these cells are immature with regard to their electrophysiological properties. Stem cells, to date, do not appear to fully express all channels at the same density and at the same proportion as occurs in human ventricular myocytes (20).

Multi-channel interactions, hERG-independent proarrhythmia mechanisms, active metabolites, or antiarrhythmic effects of a drug are not the only reasons for a lack of absolute concordance of risk estimates based on surrogate markers with actual TdP risk in a patient population. Apart from pharmacodynamic effects, TdP occurrence is influenced by many factors that define inter- and intra-individual variability (21–23). It is known that TdP occurrence heavily depends on concomitant risk factors including age, gender, electrolyte imbalance, heart rate (specifically bradycardia), and presence of structural heart disease. Also, the actual risk for an individual patient is variable with individual circadian rhythms (i.e., heart rate, serum electrolyte levels), and rhythms with a longer periodicity (related to the effects of sex hormones) can influence the individual response of the heart to drugs (24,25). Mechanisms responsible for the development of cardiac arrhythmias may be categorized according to those that modify impulse formation (such as altered normal automaticity or triggered activity caused by early or delayed afterdepolarizations) or conduction (such as that due to re-entry or re-excitation of cardiac tissue).

In humans, the most common causes of arrhythmias include myocardial ischemia, myocardial infarction, or reperfusion of a previously ischemic myocardium. These conditions can be readily reproduced in both intact and isolated hearts in many species. While the pathogenesis of arrhythmias may not appear to be relevant to drug safety, it is the mechanism(s) derived from decades of arrhythmia studies that is being used to define and explain drug-induced arrhythmogenesis. These reported mechanisms may have direct implications in the development of the CIPA paradigm and its ability to provide additional information regarding the “proarrhythmia potential” for a new drug. The mechanism(s) that have been developed will be important in the interpretation of ion channel data and the predicted changes in the action potential using suggested *in silico* models.

Abnormal impulse generation that may be responsible for induction of TdP arrhythmias may arise from oscillations in the membrane potential and has been characterized as a “triggered” rhythm (26). These triggered rhythms occur in two forms: early or late afterdepolarizations (EAD or DAD). Early afterdepolarizations interrupt either phase 2 or 3 repolarization of the AP. If these afterdepolarizations attain sufficient thresholds, they may produce triggered responses and induce single or multiple extrasystoles or polymorphic VT episodes such as TdP. The EAD is an oscillatory potential that is sensitive to frequency and often occurs at slow stimulation rates. EAD activity has been shown *in vitro* using many types of isolated cardiac tissue and various cell types including mid-myocardial cells (M-cell) (27). Induction of EAD activity can be induced by a variety of drugs that block sodium and potassium channels. When a transient depolarization occurs during phase 4 of the cardiac AP, it is termed a delayed afterdepolarization (DAD) which is vitally dependent upon the rate of the preceding action potential. Thus, the amplitudes of DADs increase with decreasing cycle lengths (28). DADs have been observed under a variety of experimental conditions, all of which have a similar end result—intracellular Ca^2+^ overload. High intracellular Ca^2+^ concentrations saturate sarcoplasmic reticulum sequestration mechanisms resulting in Ca^2+^ oscillations due to Ca^2+^-induced Ca^2+^ release (29). The ionic currents that contribute to this mechanism are not known.

For many years, there has been an effort to mathematically describe the genesis of the cardiac action potential. Primarily driven by academic research groups, there is now ongoing integration of these *in silico* research methods to the CIPA initiative. Of the many *in silico* models developed, it is the O’Hara-Rudy model that will likely form the basis for the *in silico* component of CIPA (30). The O’Hara model is a human ventricular cardiac AP model based upon data measured from over 100 undiseased human hearts. Components of the model were evaluated over the human range of physiological frequencies and include calcium *versus* voltage-dependent inactivation of L-type calcium current (*I*_CaL_); realistic kinetics for the transient outward, rapid delayed rectifier (*I*_Kr_), and inward rectifier (*I*_K1_) potassium currents along with the Na^+^/Ca^2+^ exchanger (*I*_NaCa_). The authors also examined model response to rate dependence and restitution of cardiac AP duration (APD).

### Overview of Methods and Models Used in Assessment of Drug-Induced Cardiotoxicity During Drug Development

The critical nature of cardiac liability determination and implementation of an appropriate strategy for drug safety assessment resulted in the development of three guidelines that outlined non-clinical (ICH S7A; ICH S7B) and clinical (ICH E14) testing strategies (7,31–33). The current non-clinical testing strategy includes *in vitro I*_Kr_ current assessment in heterologous mammalian cell lines expressing the human ether-a-go-go-related gene (hERG, Kv11.1) channels, which have been agreed to play a crucial role in human cardiac electrophysiology. The preclinical guidelines propose that sponsors consider other complementary models. These include a number of *in vitro* assays that have been well characterized with utility in the safety profiling of a new chemical entity (NCE). These assays include assessment of drug effects in the isolated rabbit Purkinje fiber preparation, the isolated Langendorff heart, and the isolated wedge preparation. Cardiac safety pharmacology *in vivo* methods primarily use conscious telemetered animals to assess the effects of the test item on the QT interval. Variables that are usually recorded in the dog and other non-rodent species include heart rate and the ECG. Thus, a variety of tests are used to evaluate drug safety and include effects primarily on blood pressure, heart rate, the ECG, repolarization (APD), the hERG (*I*_Kr_) ion channel, and cardiac conduction.

An additional consideration to drug effects on heart rhythm involves cardiac (ventricular) contractility. This has largely been neglected by safety scientists and has only recently become of potential interest in the cardiovascular safety milieu (34). Contractility, it is thought could initially represent a complementary readout to the current cardiovascular endpoints assessed under the core-battery ICH S7A guidance, but this could change. Several papers have recently been published that evaluate direct measures of contractility (*in vivo*), and contractility variables include left ventricular pressure (LVP) and rate of contraction and relaxation (±dP/dtmax) recorded directly via invasive catheter implantation (34,35).

Regardless of the constituents, the resultant data, in addition to data acquired from acute and/or chronic toxicology studies, are recommended to be analyzed using an integrated risk assessment (IRA) (36). The IRA is a holistic evaluation of non-clinical study results and is used because there has been no conclusion regarding which single non-clinical model could be used to accurately address issues highlighted by the regulatory guidance documents. However, the value ascribed to data derived from *in vitro* and *in vivo* studies is limited by other factors which may distort the perceived safety of a drug (37,38). These factors include differences in the pharmacokinetic and pharmacodynamic relationship between animals and humans as well as differences between species regarding metabolism and plasma protein binding as well as variability in species dependence of TdP susceptibility (39). Despite such differences, the assays currently used in non-clinical safety studies can be used to inform the planning of clinical trials. Note that the information acquired during the conduct of human trials will always surpass that of the non-clinical studies in terms of relevance.

Conduct of a clinical development program, prior to approval application, provides a rigorous assessment of the drug’s propensity to prolong the QT interval in humans. Thorough QT studies (TQT) involve quantification of the degree of the drug’s influence on cardiac repolarization in healthy volunteers as compared to placebo and a positive control (moxifloxacin). The aim of the TQT studies is to identify drugs that clinically require more attention toward the potential for development of a cardiac liability and therefore require additional ECG monitoring in subsequent clinical trials to assess arrhythmia risk in the target patient population. Although the TQT studies are informative and the best amongst the currently available methods, they may not be cost-effective and suffer from a low positive predictive value (40).

The classical approach to the assessment of proarrhythmia occurs via the so called thorough QT (TQT) study that looks for the largest excursion following a dose (a guard against hysteresis) and checks whether the upper confidence limit excludes the effect size deemed of potential clinical relevance. Using the ICH E14 guidance, the upper 90% confidence limit and a boundary of 10 ms defines the risk. However, hysteresis is rarely observed in practice, so this procedure is both inefficient in the use of available data to look for an effect and is biased toward incompletely compensating for multiplicity by taking the largest observed value amongst several time points.

Exposure-response modeling offers advantages in efficiency by combining what is known across all time points and several doses. It is also relatively simple because there is usually a linear relationship between exposure and QT for the small effects that are of interest. Indeed, such assessments have long been performed as supplements to the classical analyses of TQT studies. However, as supplementary analyses, these have not had full prespecification and are thus regarded as exploratory.

Recent work has taken a more systematic approach, with full prespecification of analytic methods, including tests for linearity. Retrospective application of formal methods of exposure-response analysis across selected studies was found to be encouraging, but Food and Drug Administration (FDA) insisted on a prospective study, too, and named a small set of drugs and doses with peak effects in the neighborhood of 10 ms to be assessed (41)

The conducted study was a three-period, third-party blinded, randomized, placebo-controlled study in 20 healthy volunteers. The design was planned to be similar to a single ascending dose (SAD) phase 1 study with the primary objective to estimate the effect of the drugs on the QTc interval using ER analysis. Each subject undergo three treatment periods. An incomplete block design was used what resulted in each study drug being administered to nine subjects and placebo to six subjects in separate periods. The design, sample size, and statistical approach is intended to result in similar power to exclude clinically relevant QTc effects as a standard SAD FIM study (41).

Exposure-response methods have now been established in order to provide a more efficient assessment of the QT interval, making such an assessment potentially a part of early phase clinical studies where the highest doses are likely to be utilized, and obviating conduct of a dedicated, expensive TQT study. However, this methodology still makes use of a biomarker whose correlation to proarrhythmic risk is not absolute.

To address the deeper problem, we can potentially make use of fundamental understanding of the mechanism by which torsade-like arrhythmias are generated, and the ability to assess *in vitro* drug effects on the machinery derived from humans.

Torsade occurrence requires two things. The first is a relative failure of the cardiac myocyte to execute the repolarization phase of the action potential. Following the upstroke and plateau phases, the myocyte should re-establish polarity, allowing the trigger to be reset for the next action potential at its expected time. During this time, the repolarization forces, in the form of outward currents, must be vigorous enough to suppress any residual tendency for regenerative activity. Thus, inadequate outward current activity during this time allows inward, depolarizing current to get the upper hand leading to “early afterdepolarizations” (EADs), essentially, new action potentials happening long before they are due.

As long as these EADs happen more or less throughout the myocardium in synchrony, they are generally harmless. At least, following one bad beat, things are likely to settle back to normal activity (42,43). This is why patients with intrinsic or drug-induced problems with repolarization can live decades without a fatal arrhythmia. So, the second criterion for setting up an arrhythmia is some degree of heterogeneity in the heart. If all parts of the heart are not working in synchrony, say, because of regional ischemic disease, scarring due to an healed infarct, etc., the errant action potential has some place to conduct and then is able to back-propagate and re-excite cardiac tissue into what becomes a circuitous activity (42). During such an event, the usual muscular coordination is lost and the pumping action of the heart is interrupted. Such effect has been widely studied and discussed from various perspectives (44).

**Fig. 1:**
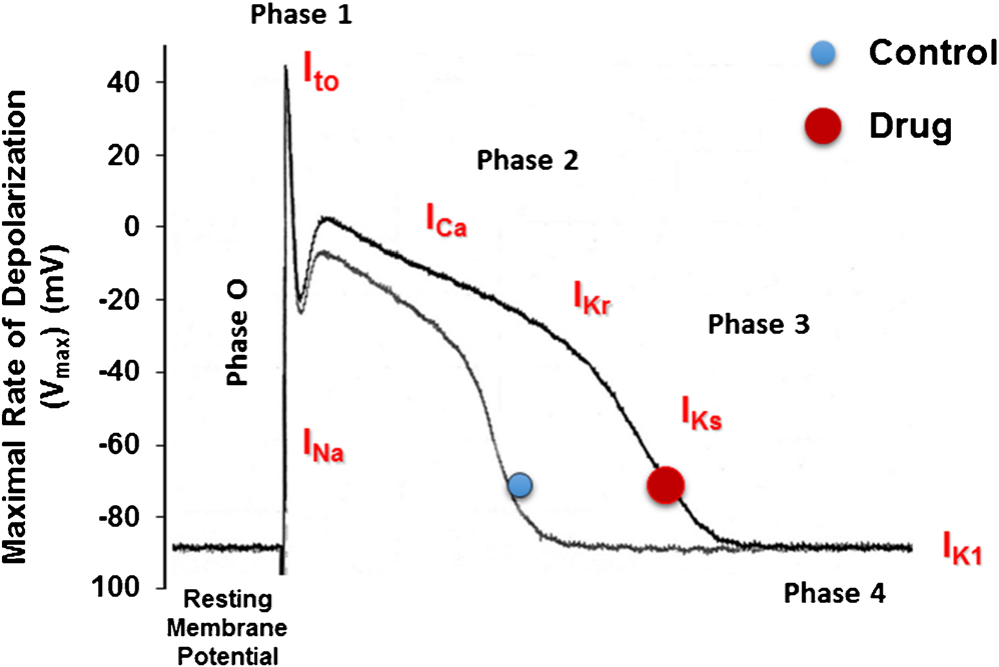
A cardiac action potential that outlines the major currents involved in depolarization and repolarization of the cell membrane. The cardiac action potential (*AP*) conventionally consists of several phases (0–4) with a duration of ∼300 ms. Phase 0 corresponds to membrane depolarization (Na^+^ influx thru *I*_Na_ channels) while phase 1 shows the early rapid repolarization of the membrane due to activation of the transient outward (*I*_to_) K current. Phase 2 is the plateau of the AP (due to a reduction in Na^+^ influx) and an increase in Ca^2+^ influx (thru *I*_Ca_ channels) while phase 3 shows membrane repolarization resulting from the coordinated opening and closing of many different K^+^ channels such as the rapid (*I*_Kr_) and slow (*I*_Ks_) components of the delayed rectifier K channel. Phase 4 corresponds to the resting membrane potential and is maintained by the inward rectifier (*I*_K1_) channel. The effects of a drug that produces a prolongation in the AP by blockade of *I*_Kr_ is shown (drug (*circle*))

Although we can find evidence for the electrical uncoupling in the heart and can model its effects to gain insights into proarrhythmic mechanisms, fully characterizing a patient’s intrinsic risk in this regard lies outside what is technically feasible today. Judging when an arrhythmia will occur and in whom is not possible. Judging which drugs are likely to create the conditions that give rise to EADs is, on the other hand, quite feasible (see above). Cardiac myocytes have multiple ion channel types (see Fig. 1), and the human channels of each type can be studied in isolation in cells overexpressing single types. Each channel type can have the effect of a drug assessed *in vitro* under voltage clamp conditions, and high-throughput systems permit replicate experiments, exposure-response characterization, etc. to be performed at modest cost. If you know the channel densities in human cardiac myocytes, you can then reconstruct the human cardiac myocyte action potential, as it is influenced by time-varying drug exposure, *in silico*, and look for the propensity to produce EADs. That is, at the level of the individual human cardiac ventricular myocyte, the process of developing an action potential is completely understood, and drug effects can be completely characterized.

Because this technology assesses drug effects across all, or most, of the set of cardiac ion channel types, it should have the ability to detect drugs with isolated adverse effects on repolarization and to differentiate those drugs from the ones with mixed effects on depolarizing and repolarizing forces, resulting in less net risk.

In practice, there are some uncertainties. For example, you cannot be sure that the voltage clamp protocol you used will always capture the effect of a drug on the ion channel, because that effect may depend on some aspect of the channel’s history that your protocol did not account for. There can also be effects of drugs that are not the result of direct interaction with the channel protein. It is, therefore, of interest to augment such a channel-based assay with some information from a more integrated (and thus less easily characterized) system. Two such candidates are being explored.

Ventricular myocytes can be obtained directly from adult human ventricles, but sufficient availability cannot yet be guaranteed by vendor sources (45). Human embryonic or pluripotent stem cells can be induced to form ventricular myocytes, which can be cultured in vast numbers, and these are available from numerous commercial sources. While they do not currently replicate the electrophysiological phenotype of myocytes from the adult human heart, they can be used, once correctly engineered, to provide some assessment of drug effects that might be missed by the use of the hERG channel-based assay alone.

Another approach to attaining supplementary information on drug effects is to return to the human ECG. The upstroke of the action potential is reflected in the QRS interval of the ECG, and repolarization is represented by the QT interval. Drugs with a variety of known channel effects have predictable effects on the morphology of the ECG waveform, and understanding these relationships and what is meant by their changes can help make predictions about underlying channel effects (with potential clinical implications) of novel drugs.

### *In Vitro*–*In Vivo* Extrapolation of a Drugs’ Proarrhythmic Effect—From High-Throughput to Rare Case Analysis

All the abovementioned methods focus on a single compound or at most in combination with metabolites when an *in vivo* system is in use, and idealistic (or non-realistic) conditions which are not complimentary with the real life situation where the drug of interest is used. Even a cursory analysis of the literature-reported TdP cases suspected to be drug-triggered depicts one element which seems to be overlooked, namely the influence of external factors.

Multiple elements can be listed as external factors, and the list below does not cover all possible components:

1. Concomitant drugs and the pharmacokinetic (PK) and pharmacodynamics (PD) drug–drug interactions
2. Food and other environmental factors
3. Demographic and physiological parameters and their drug-triggered modification
4. Genetic factors and comorbidity

Apart from a direct influence on the drug affinity to the channels, all of the above-listed elements can modify drug pharmacokinetics. It has been widely proven that the variability of physiological parameters directly modify drug pharmacokinetics and exposure (46–48). Therefore, cardiac risk should ideally be assessed at the level of the individual patient and also account for non-drug-related parameters potentially triggering serious health-threatening situations. Examples of such situations can be highlighted amongst the drugs which are currently on the market or were withdrawn due to non-acceptable cardiac risk. To support such a statement, a brief, non-exhaustive analysis and description of the commonly known proarrhythmic drugs has been performed and discussed to draw attention to the role of these non-drug parameters.

Cisapride is often given as an exemplary non-cardiology drug associated with the risk of TdP (49). Cisapride, a serotonin 5-HT_4_ receptor agonist, was developed in 1980 as a prokinetic agent that increased gastrointestinal motility. It has also been used for the treatment of gastroesophageal reflux disease. *In vitro* studies revealed that it was a potent *I*_Kr_ current inhibitor with *in vitro* measured IC_50_ values in the range of 4.3–100 nM depending on the study settings (50). However, it has other ion channel properties that include late calcium (*I*_CaL_) current inhibition with IC_50_ values in the micromolar range (51). According to all known classification schemes including those developed by Redfern, Mirams, and Crediblemeds (https://www.crediblemeds.org/), it is a drug associated with a high propensity for proarrhythmic risk (9,52,53). This assignment of risk is based on case reports of side effect including TdP and other types of arrhythmias, especially when the medication was taken concomitantly with other medications or in patients with certain underlying conditions predisposing them to arrhythmias. Wysowski *et al*. analyzed the post-marketing reports of QT prolongation and ventricular arrhythmia associated with cisapride (54). From 1993 until 1999, while being marketed in the USA, the FDA received 341 individual patient reports of multiple heart-related conditions including 117 associated with QT prolongation, 107 with TdP, 16 with polymorphic ventricular tachycardia, and 27 with ventricular tachycardia. Eighty (23%) of the 341 patients died. The authors concluded that in most cases, an arrhythmia occurred in the presence of additional, complex risk factors including the presence of other drugs and/or variant medical conditions. Among other strictly contraindicated factors that were listed include concomitant use of CYP3A4 enzyme inhibitors, serious heart conditions, electrolyte disorders, and overdose. A similar situation was reported in the case study provided by Hussain and Ghazal (55). These authors report the medical situation of a 36-year-old woman presented to the emergency room after 3 days of abdominal pain, fever, nausea, and vomiting preceded by a Caesarean section. She was treated by multiple drugs including antiarrhythmic drugs and cisapride which were considered as the reasons for the developed ventricular tachycardia which degenerated to frequent episodes of TdP. After testing multiple factors, the authors concluded that the observed TdP might not be solely due to drug-induced QT interval prolongation. Other factors discussed by the attending clinicians include those caused by hypokalemia resulting from repeated vomiting and poor nutritional intake and metabolic drug–drug interactions which altered cisapride exposure. Cisapride was withdrawn from many global markets in 2000 but remains available for use in some EU countries to specific patients under strict black-box restrictions. Use by these specific patients requires that both health care providers and patients receiving cisapride are familiar with this complication associated with use and that all parties understand and comply with specific recommendations outlined and required for use.

Trimebutine, a drug used to regulate motility in the gastrointestinal tract via an agonist effect on peripheral μ-, κ-, and δ- opiate receptors and modulation of gastrointestinal and extragastric peptide release (i.e., motilin, vasoactive intestinal peptide, gastrin, and glucagon), is provided as an example distinctly different from that of cisapride. Trimebutine is a weak inhibitor of *I*_Kr_ currents in guinea pig ventricular myocytes as described by Morisawa and colleagues, with negligible effects, even at concentrations much higher than those in clinical use (56). As may be expected, based on such studies, trimebutine is classified as a drug without known TdP risk (53). Surprisingly, a Eudravigilance system (i.e., a data processing network and management system for reporting and evaluating suspected adverse drug reactions) query resulted in the finding of two records that involve cases of TdP associated with its use (57). Both cases were reported by health care professionals which increases their potential credibility, but interestingly, both cases concern effects on elderly patients (65–85 years of age). Such reports do not provide a complete set of information and obviously cannot be used as strong evidence of potential risk but can be used in signal generation. Additionally, a quick literature search (scholar.google.com) found one report where significant QT interval prolongation and monomorphic ventricular tachycardia was observed with high doses of trimebutine (58). Monomorphic ventricular tachycardia, as opposed to the polymorphic variant, is less dangerous and more easily manageable but can degenerate to a polymorphic form including TdP. The authors conclude that while there may be a causal association between the occurrence of arrhythmia and the use of high doses of trimebutine, it is only probable. However, this may be sufficient to warrant further review to quantify drug and non-drug-related triggering factors (in this case—sex, age, and plasma electrolytes).

In most cases, the clinically observed effect is likely the consequence of the multiple actions the drug impart on varying physiological systems. For some drugs, metabolites contain inhibitory activity against ionic currents; therefore, knowledge about their pharmacokinetics and pharmacodynamics allows for a better prediction of the cardiac effects and clinical data interpretation. In 2010, after 13 years of a presence on market, the US FDA issued a safety announcement regarding Anzemet (dolasetron mesylate—an antiemetic 5-HT_3_ receptor inhibitor) use, informing patients and health professionals that the injectable form of Anzemet should no longer be used to prevent nausea and vomiting associated with cancer chemotherapy (CINV) in pediatric and adult patients (59,60). Such a decision was undertaken after review of dolasetron-induced TdP cases (61). The drug can still be used in postoperative nausea and vomiting prophylaxis and treatment because lower doses are used for these indications. Dolasetron is rapidly metabolized to a reduced form of hydrodolasetron (MDL 74,156) by carbonyl reductase, an enzyme widely distributed in human tissues (62,63). In view of this manner of metabolism, dolasetron is considered as a prodrug that is converted to hydrodolasetron, which is believed to be responsible for the majority of clinical activity (64,65). Additionally 5′-OH and 6′-OH metabolite derivatives are considered as carrying partial activity. When given orally, dolasetron plasma concentration is in most cases undetectable and its pharmacological activity is negligible, although after intravenous injection both active moieties are present at the site of action and trigger potential cardiac effects. Orally taken, dolasetron formulations are still in use for all indications and considered as safe (66). There was however a case study published where a massive orally taken dolasetron dose (2000 mg, p.o.) was taken. The patient’s ECG showed first-degree heart block along with non-specific intraventricular conduction delay and a prolonged QTc interval (67).

The above given example indicates that the route-dependent kinetic actions of drugs should be considered during drug safety analysis and that a combination of the active substances (i.e., parent and metabolites) rather than a single entity (i.e., the parent molecule) should be studied for safety purposes. This is done depending upon the levels of the metabolites in the plasma (>10%) and whether they can be synthesized and tested alone. Similarly, toxicology species are assessed for parent and metabolite PK parameters to ensure that adequate exposure of parent and metabolites occurs during long-term toxicity assessments. There are multiple examples of drug–drug interactions at the pharmacokinetic and pharmacodynamic level which trigger potential toxic effects with terfenadine and ketoconazole being probably the most well-known examples (68). Consequences of the latter are relatively easy to predict as ketoconazole-driven CYP3A4 inhibition and subsequent blockade of terfenadine metabolism resulted in substantial increases in terfenadine blood concentrations in combination with potent inhibition of the *I*_Kr_ current resulted in QT interval prolongation and precipitation of TdP arrhythmias. At the daily routine level, what is probably most important to assess is the non-linear effect of the drug combination. Such a situation was described for droperidol and ondansetron where, despite of lack of exposure modification after concomitant dosing, the QT interval was prolonged, but the observed prolongation was not proportional to the QT prolongation observed for two drugs given separately (69).

Considering the complexity of the above-listed phenomena, their thorough analysis during the conduct of clinical trials would be very challenging, if not impossible. What’s more, clinical trial characteristics, namely a relatively small cohort, homogeneity of the included individuals, short period of drug exposure, rare drug–drug and drug–environment interaction analysis, could contribute to relatively poor prediction of rare cases observed in subsequent clinical studies. The solution might be the development of a relatively tight safety margin for the analyzed TdP risk surrogate as was proposed in the ICH E14 guideline. There is however a cost connected with that as it might, and probably has, provoked genesis of a high percentage of false positives and thus a high clinical attrition rate of many drug candidates (1). As was mentioned above, this was one of the reasons for the inception of a new cardiac safety testing paradigm discussion and likely introduction. An inevitable component of this new paradigm is *in silico* methods which should became a vital element of cardiac safety testing. These include various approaches, starting from screening methods (QSAR-based models), up to the utilization of the biophysically detailed cardiac myocyte models (15,70). The latter techniques vary with regard of the level of complexity of the mathematical description of the cardiac physiology at the ion channel (Hodgkin-Huxley or Markovian notation) and cell level (single cell up to the three-dimensional heart structure) (71,72). Such methods offer the possibility to incorporate variability of either stochastic or deterministic nature (73,74). This can further allow for the drug cardiac safety analysis at the population level and quantitative assessment of the combination of drug and non-drug-related parameters (36,75,76).

Some elements should include the need for proper exposure quantification. The effect at the clinical level is related to the concentrations of the tested substances. However, plasma drug concentration (which is the most common effective concentration surrogate) can be imperfect as it may vary from that in the tissues. Therefore, more suitable effector concentration methods should be considered whenever available, possibly in the places where drug might meet cardiac ion channels (i.e., pericardial fluid, heart cell extracellular matrix, and cardiomyocyte cytoplasm). Something that is impossible in clinical practice can be potentially incorporated via the application of a physiologically based pharmacokinetic (PBPK) modeling and simulation approach.
